# Molecular Characterization and Phylogenetic Analysis of Dengue Fever Viruses in Three Outbreaks in Tanzania Between 2017 and 2019

**DOI:** 10.1371/journal.pntd.0011289

**Published:** 2023-04-26

**Authors:** Maria Ezekiely Kelly, Frank Msafiri, Muna Affara, Florian Gehre, Nyambura Moremi, Janeth Mghamba, Gerald Misinzo, Thorsten Thye, Wangeci Gatei, Toni Whistler, Agricola Joachim, Nsiande Lema, Gilberto A. Santiago

**Affiliations:** 1 National Public Health Laboratory, Ministry of Health, Dar es Salaam, Tanzania; 2 Department of Microbiology and Immunology, Muhimbili University of Health and Allied Sciences, Dar es Salaam, Tanzania; 3 Bernhard Nocht Institute for Tropical Medicine, Infectious Disease Epidemiology Department, Hamburg, Germany; 4 East African Community Secretariat, Health Department, Arusha, Tanzania; 5 Department of Epidemiology, Ministry of Health, Dodoma, Tanzania; 6 SACIDS Foundation for One Health, Sokoine University of Agriculture, Morogoro, Tanzania; 7 Centers for Disease Control and Prevention, Division of Global Health Protection, Atlanta, Georgia, United States of America; 8 Tanzania Field Epidemiology and Laboratory Training Program, Dar es Salaam, Tanzania; 9 Centers for Disease Control and Prevention, Division of Vector-Borne Diseases, Dengue Branch, San Juan, Puerto Rico, United States of America; University of Florida, UNITED STATES

## Abstract

**Background:**

Dengue is a disease of public health interest, and Tanzania experienced major outbreaks in 2014 and 2019. Here, we report our findings on the molecular characterization of dengue viruses (DENV) that circulated during two smaller outbreaks (2017 and 2018) and one major epidemic (2019) in Tanzania.

**Methodology/Principal findings:**

We tested archived serum samples from 1,381 suspected dengue fever patients, with a median age of 29 (IQR:22–40) years, referred to the National Public Health Laboratory for confirmation of DENV infection. DENV serotypes were identified by reverse transcription polymerase chain reaction (RT-PCR), and specific genotypes were identified by sequencing the envelope glycoprotein gene and phylogenetic inference methods. DENV was confirmed in 823 (59.6%) cases. More than half (54.7%) of patients with dengue fever infection were males, and nearly three-quarters (73%) of the infected individuals were living in Kinondoni district, Dar es Salaam. DENV-3 Genotype III caused the two smaller outbreaks in 2017 and 2018, while DENV-1 Genotype V caused the 2019 epidemic. DENV-1 Genotype I was also detected in one patient in 2019.

**Conclusion/Significance:**

This study has demonstrated the molecular diversity of dengue viruses circulating in Tanzania. We found that contemporary circulating serotypes did not cause the major epidemic of 2019 but rather due to a serotype shift from DENV-3 (2017/2018) to DENV-1 in 2019. Such a change increases the risk for patients previously infected with a particular serotype to develop severe symptoms upon potential re-infection with a heterologous serotype due to antibody-dependent enhancement of infection. Therefore, the circulation of serotypes emphasizes the need to strengthen the country’s dengue surveillance system for better management of patients, early detection of outbreaks, and vaccine development.

## Author summary

In Tanzania, dengue has been reported since 2010, and different DENV serotypes have been documented to circulate in the country, causing two large epidemics (2014 and 2019). Rapid diagnostic tests have been the country’s preferred laboratory tool for diagnosing dengue. Confirmatory tests, such as polymerase chain reaction (PCR) and genomic sequencing, are unlikely to be available for all samples collected during an outbreak, mainly due to resource constraints. Consequently, national surveillance of DENV cannot detect circulating serotypes and genotypes, especially during epidemic transmission periods. This information is critical to inform and support effective disease control interventions and prepare for future outbreaks. This study analyzed all the collected samples between 2017 and 2019 and referred to the National Public Health Laboratory (NPHL) for molecular characterization. Here we report the serotypes and phylogenetic characteristics of DENV from 2017 to 2019 circulating in Tanzania.

## Introduction

Dengue is a mosquito-borne viral infection widespread throughout the tropics [1]. The global incidence of the disease has increased dramatically in the past few decades, with an estimated 100–400 million new infections occurring each year [1]. About half of the world’s population is at risk of infection [2], whereby 96 million people are symptomatic [3]. Asia accounts for two-thirds of the global burden, and Africa for 16% of the global cases, though DENV transmission in Africa remains largely underreported [3].

Dengue virus (DENV) is an enveloped positive-sense ribonucleic acid (RNA) virus that belongs to the *Flaviviridae* family [4]. There are four serotypes of DENV: DENV-1, DENV-2, DENV DENV-3, and DENV-4 [5] that are closely related antigenically, sharing 65–70% of the amino acid sequence [6,7]. The genome of DENV is approximately 11 kb long and encodes 10 proteins, comprising of three structural proteins and seven nonstructural (NS) proteins [8]. The envelope protein has been widely used in DENV sequencing studies because of its sequence variability, influenced by its immunological function and environmental exposure that select for variants arising from mutations during replication [9–11]. DENV is transmitted primarily through the bites of infected female *Aedes aegypti* mosquitoes [12]. The mosquito is a predominantly daytime feeder that thrives in warm and humid climate. The female *Aedes aegypti* lay multiple batches of eggs in water-filled containers that hatch into larvae during favorable conditions, especially at high temperatures [13]. Dengue has been circulating in Africa since the 19^th^ century [14]. To date, the disease is endemic in 34 African countries, with DENV-2 being the predominant serotype [15,16]. Being in the endemic region, Tanzania has experienced two major dengue outbreaks; in 2014 and in 2019. Despite the two epidemics, the disease burden is still unknown due to poor surveillance [17–19]. According to a study conducted in northern Tanzania in 2013 and 2014 investigating febrile illnesses other than malaria, it was reported that dengue and chikungunya are overlooked since most facilities do not have sufficient testing capacity or access to rapid tests for their detection [20]. Since there is no laboratory-based surveillance or routine diagnostic testing for DENV at most health facilities in the country, dengue is often misdiagnosed as other acute febrile illnesses [20].

Tanzania has 31 regions [21], and dengue has been reported in several regions; Dar es Salaam, Tanga, Morogoro, Kilimanjaro, Pemba, Zanzibar, Manyara, Kigoma, Njombe, Mwanza, Dodoma, Mbeya, Iringa, and Zanzibar [22,23]. The first major dengue outbreak was caused by the cosmopolitan genotype of DENV-2, which infected more than 1,018 individuals in 2014 [24]. In 2019, a second major epidemic occurred in Tanzania, resulting into more than 6,917 infected cases and 13 deaths. DENV-1 was identified as the cause of the 2019 outbreak [18,25]. The increase in number of cases may signal the evolution of the virus as it spreads in mosquito and humans populations in the country [26]. In this study, we report the serotypes, genotypes, and geographic distribution of DENV strains that caused the three outbreaks between 2017 and 2019.

## Methods

### Ethics statement

The Senate Research and Publications Committee of Muhimbili University of Health and Allied Sciences (MUHAS) approved the waiver of informed consent and the study protocol with Ref No. DA.282/298/01.C/400. Permission to use public samples was obtained from the Chief Medical Officer of the Tanzania Ministry of Health.

### Study design and setting

We conducted a cross-sectional study involving 13 regions that experienced dengue outbreaks in Tanzania between 2017 and 2019. The study used archived serum samples collected from patients presenting with febrile illness at various healthcare facilities in the 13 regions. Samples were collected aseptically using plain red top vacutainer, processed to obtain serum and then shipped to the National Public Health Laboratory (NPHL) for confirmation of DENV infection using reverse transcription polymerase chain reaction (RT-PCR). Samples were stored at -80°C until testing. All the archived samples at NPHL from dengue suspect cases were included in this study (Table 1). Social demographic information was obtained from the line lists and laboratory request forms from different hospitals that submitted these samples.

**Table 1:** Distribution of dengue samples per region from 2017–2019.

Regions N = 1,381	Samples collected	Percentage
Dar es Salaam	1288	93.3
Tanga	69	5
Kilimanjaro	6	0.43
Coast	8	0.58
Mbeya	2	0.14
Rukwa	1	0.07
Singida	1	0.07
Songea	1	0.07
Bukoba	1	0.07
Dodoma	1	0.07
Mtwara	1	0.07
Mwanza	1	0.07
Zanzibar	1	0.07

### Dengue virus RNA extraction

All the samples were stored at -80°C and archived until ready to test, then thawed before nucleic acid extraction. Extraction of viral RNA was performed using QIAamp Viral RNA Mini Kit (QIAGEN, Hilden, Germany), following manufacturer’s recommended instructions.

### Dengue virus typing

Molecular confirmation of DENV infection was performed by using US CDC DENV-1-4 real-time RT-PCR Multiplex Assay (CDC, Atlanta, USA). The kit was obtained from the US CDC Dengue Branch. The assay was performed in multiplex on ABI 7500 real-time PCR System thermocycler (Applied Biosystems, Foster City, CA, USA), as described in the package insert.

### Serotype-specific PCR amplification and sequencing

Partial genome sequencing of DENV was conducted as described previously in Santiago et al. [27]. The DENV envelope glycoprotein (E protein) coding sequence (1,485 bp) was used as the target for amplification and sequencing by using one set (forward and reverse primer) of serotype-specific oligonucleotides (see Table 1 in [27]). All amplification reactions were carried out in a 96-well conventional GeneAmp PCR system 9700 thermocycler (Applied Biosystems, Foster City, CA). To visualize PCR products, 5 μL of each PCR reaction were mixed with 2 μL of 6x DNA gel loading dye and loaded on to a 1% UltraPure Agarose gel (Invitrogen, CA, USA) along with DNA ladder (Quick-load 1kb Plus, New England Biolabs). Agarose electrophoresis gels were stained with GelRed (Biotum, USA) before visualization under UV transilluminator.

The PCR amplicons were purified using ExoSAP-IT PCR product cleanup reagent (Applied Biosystems, Foster City, CA), as per manufacturer instruction. The purified PCR product was sequenced using 8-capillary Sanger sequencing reactions per sample with 8 serotype-specific sequencing primers (4 forward and 4 reverse reactions, see Table 2 in [27]) with Big-Dye terminator V3.1 ready reaction cycle sequencing kit (Applied Biosystems, Foster City, CA) per manufacturer’s guideline on GeneAmp PCR system 9700 thermocyclers (Applied Biosystems, Foster City, CA).

Finally, the purification of the cycle sequencing product was performed using BigDye XTerminator purification kit (Applied Biosystems, Foster City, CA). Sequencing was performed on 3500 XL genetic analyzer (Applied Biosystems, Foster City, CA). Sequence trace files and contig assembly analysis were performed using Sequencher software version 5.4.6. The consensus sequences were analyzed with BLAST (https://blast.ncbi.nlm.nih.gov/) to confirm sequence identity and retrieve similar sequences for phylogenetic analysis.

### Phylogenetic tree construction

Phylogenetic analyses with the DENV E gene sequences generated by this study were conducted using at least the top 10 sequences with high similarity resulting from BLAST available in GenBank. Multiple sequence alignments of study sequences and reference sequences for each serotype from different geographical regions were generated with Clustal W in MEGA X software [28]. For genotyping analyses, additional sequences with high similarity from different DENV studies, including sequences of contemporary geographic-temporal origin, were included to maximize the genotypes’ representation and the sequence’s geographic-temporal origin. Phylogenetic trees were inferred using the maximum likelihood method in IQ-TREE software (http://www.iqtree.org/) version 1.6.12 with 1,000 bootstrap replicates for node support test. The level of bootstrap support considered to be significant was 75%. The best fitting trees were visualized using Interactive Tree of Life (iTOL) version 6.7 web-based software (https://itol.embl.de/). The genotypes were identified using The Genome Detective Virus Typing Tool available online (https://www.genomedetective.com/app/typingtool/dengue/). Quantum Geographic Information System (QGIS) version 3.24.1 was used to draw the map of dengue cases between 2017 and 2019 in Tanzania. All the sequences from this study were uploaded to the GenBank database (32 strains for DENV-3: OM920035—OM920066; and 341 for DENV-1: OM920075—OM920415). The sequences are listed in S1 and S2 Tables.

### Statistical analysis

Data were analyzed by using STATA version 15. Frequency and proportions were used to analyze categorical variables while median with interquartile range (IQR) was used to summarize continuous variables.

## Results

### Socio-demographic characteristics of patients with dengue infection

Of the 1,381 samples tested, 823 (59.6%) were confirmed dengue positive by RT-PCR; a median age of 29 years (IQR:22–40). None of the patients tested reported having dengue hemorrhagic fever (DHF) or dengue shock syndrome (DSS). DENV infection were 450 (54.7%) for males with the highest proportion of dengue observed in patients aged 25–34 years, as shown in Table 2.

**Table 2:** Socio-demographic characteristics of patients with dengue infection in Tanzania from 2017 to 2019.

Characteristics N = 823	Number of positive cases	Percentage
Year		
2017	16	80.0
2018	40	19.0
2019	767	66.7
Gender		
Male	450	54.7
Female	373	45.3
Group age (years)		
≤ 5	39	4.7
6–14	63	7.7
15–24	180	21.9
25–34	241	29.3
35–44	142	17.3
45–54	97	11.8
55+	61	7.4

### Geographical distribution of dengue cases in Tanzania from 2017–2019

**Fig 1:**
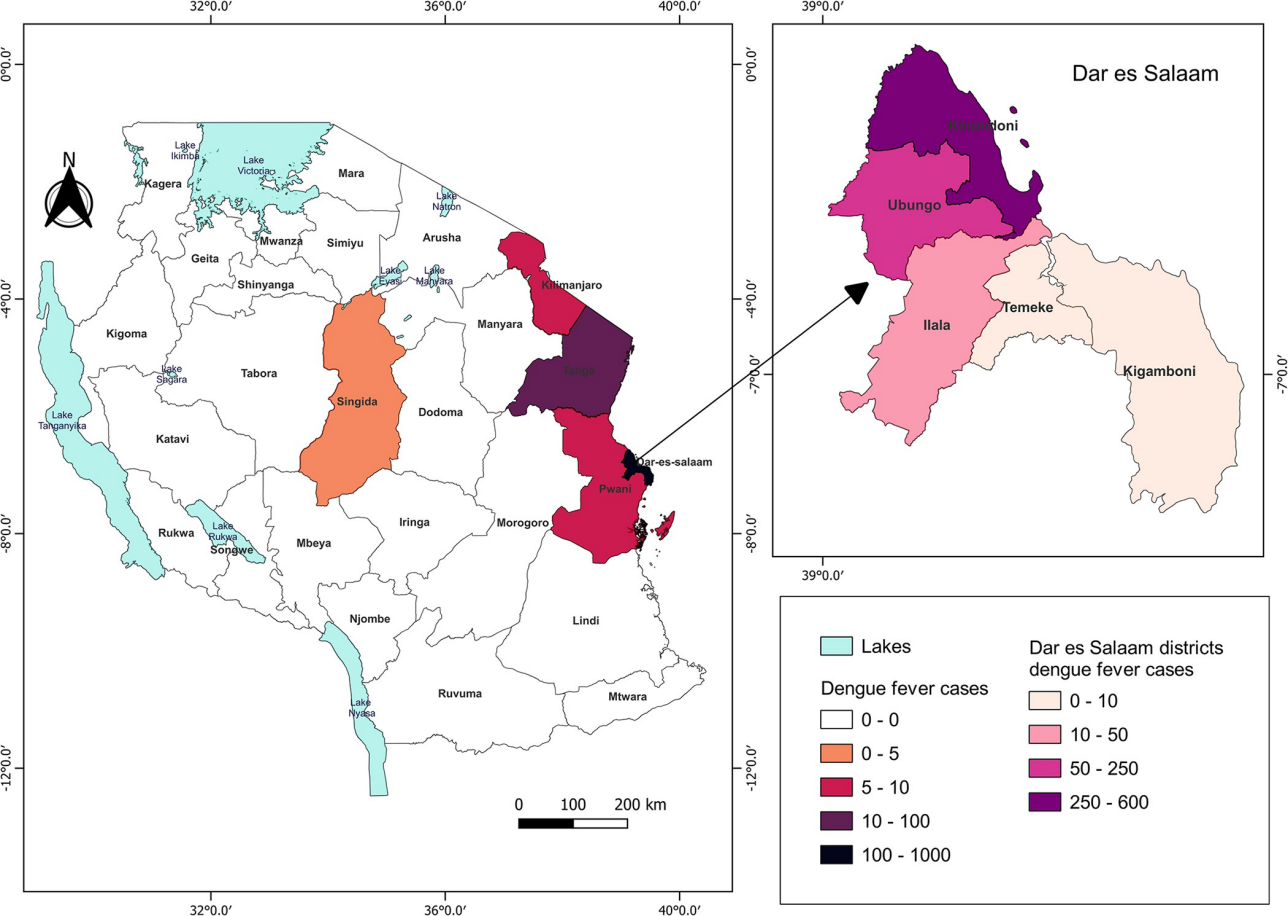
Distribution of dengue fever cases in Tanzania from 2017 to 2019. The map shows 26 Tanzania mainland administrative regions. The five color-coded regions show dengue fever cases distribution between 2017–2019. Map created with QGIS 3.24.1 All shape files are openly available sources (https://www.nbs.go.tz/index.php/en/census-surveys/gis/385-2012-phc-shapefiles-level-one-and-two). The shapefiles were made based on the 2012 population and housing census, but in this study, the shapefile has been modified to capture all the regions and district information.

The majority of cases were located in Dar es Salaam, residents from the regions of Kinondoni 552 (73%), Ubungo 147 (19.4%), Ilala 47 (6.2%), Temeke 7 (0.9%), and Kigamboni 3 (0.4%), with a total case count of 756. The other regions with reported cases were Tanga 53 cases, Coast 7 cases; Kilimanjaro 6 cases, and Singida had only one positive confirmed case as shown in Fig 1.

### Distribution of DENV serotypes

Only two serotypes were circulating in Tanzania between 2017 and 2019. In 2017 and 2018, DENV-3 was the cause of the sporadic cases (n = 56). The epidemic in 2019 was caused by DENV-1 (n = 767). We did not detect the co-circulation of DENV serotypes within the same period in this study.

### Distribution of DENV genotypes

Out of 823 DENV positive samples, 423 (51.4%) were sequenced successfully (DENV-3 n = 32, DENV-1 n = 391). We inferred phylogenetic trees to identify circulating DENV-1 and DENV-3 genotypes and confirmed our results with the web-based DENV typing tool (https://www.genomedetective.com/app/typingtool/dengue/). Both analysis methods classified all DENV sequences from 2017 and 2018 as DENV-3 genotype III. Our phylogenetic analysis classified 99.7% (390/391) DENV-1 sequences from 2019 as genotype V and 0.3% (1/391) of the DENV-1 sequences as genotype I.

### Phylogeny of DENV-3 from 2017 and 2018

**Fig 2:**
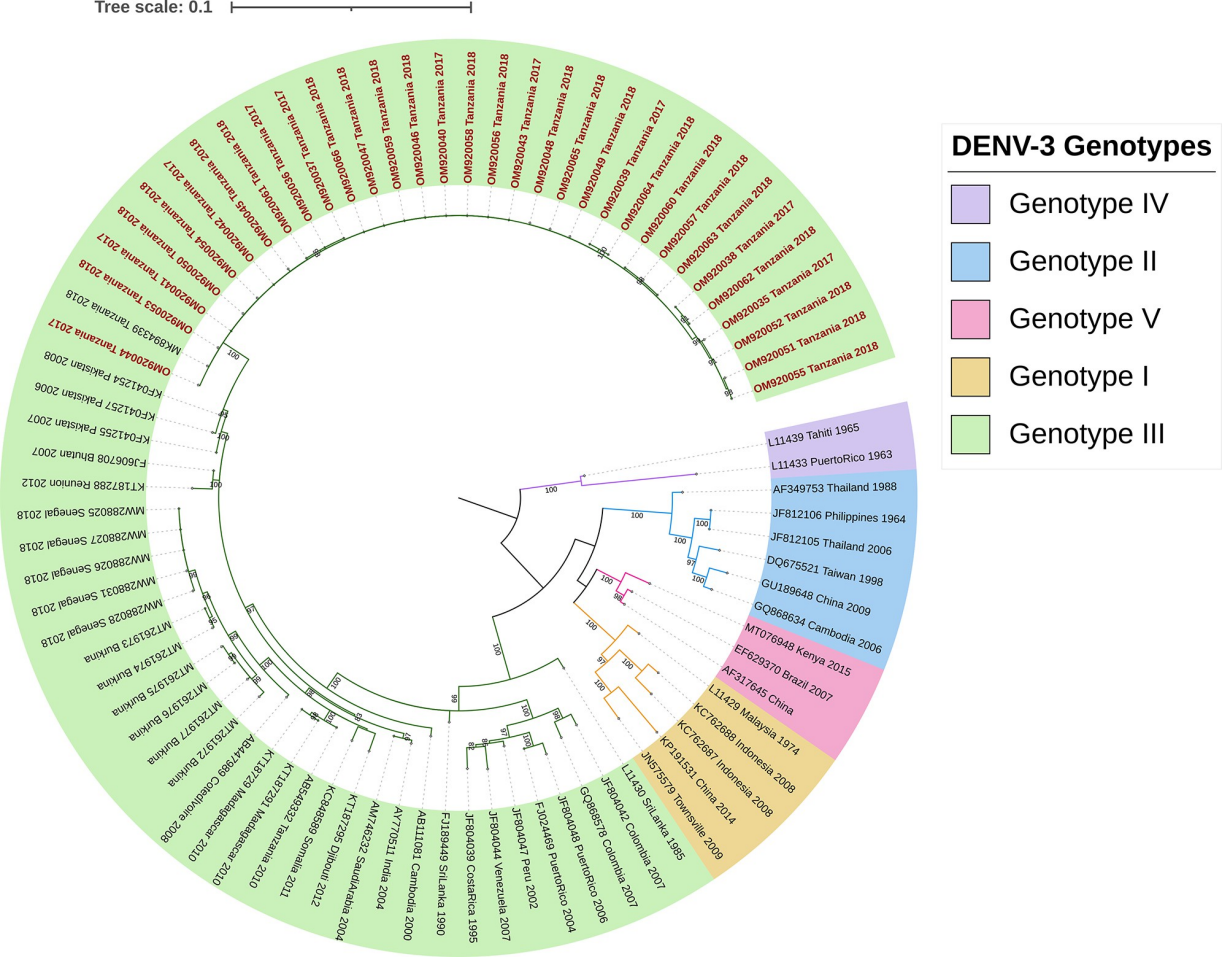
Genotyping of DENV-3 circulating in Tanzania 2017–2018. Maximum likelihood phylogenetic tree reconstructed with the 32 DENV-3 sequence generated by this study and 40 additional sequences from GenBank to provide genotype reference and geographic-temporal context. The tree was rooted at midpoint. pink, blue, green, gold and purple represents genotypes V, II, III, I, and IV, respectively. Tanzanian sequences (OM920035—OM920066) are in red. Contextual sequences are labeled with GenBank accession number, country of origin, and year of isolation.

The nucleotide sequence of the envelope gene region of all 32 DENV-3 samples (10 sequences from 2017 and 22 sequences from 2018) was used for phylogenetic analysis. Fig 2 shows the 2017 and 2018 Tanzanian DENV-3 sequences clustered together into a monophyletic lineage within the genotype III group, demonstrating close phylogenetic relatedness. The 32 sequences showed high similarity between them, and the cluster is closely associated with viruses identified in Pakistan between 2006 and 2008, with 95% bootstrap node support. One of the sequences, MK894339_Tanzania_2018, was isolated from a traveler returning to Japan from Tanzania. We did not detect distinctive clusters separating 2017 from 2018 sequences.

### Phylogeny of DENV-1 from the 2019 epidemic

**Fig 3:**
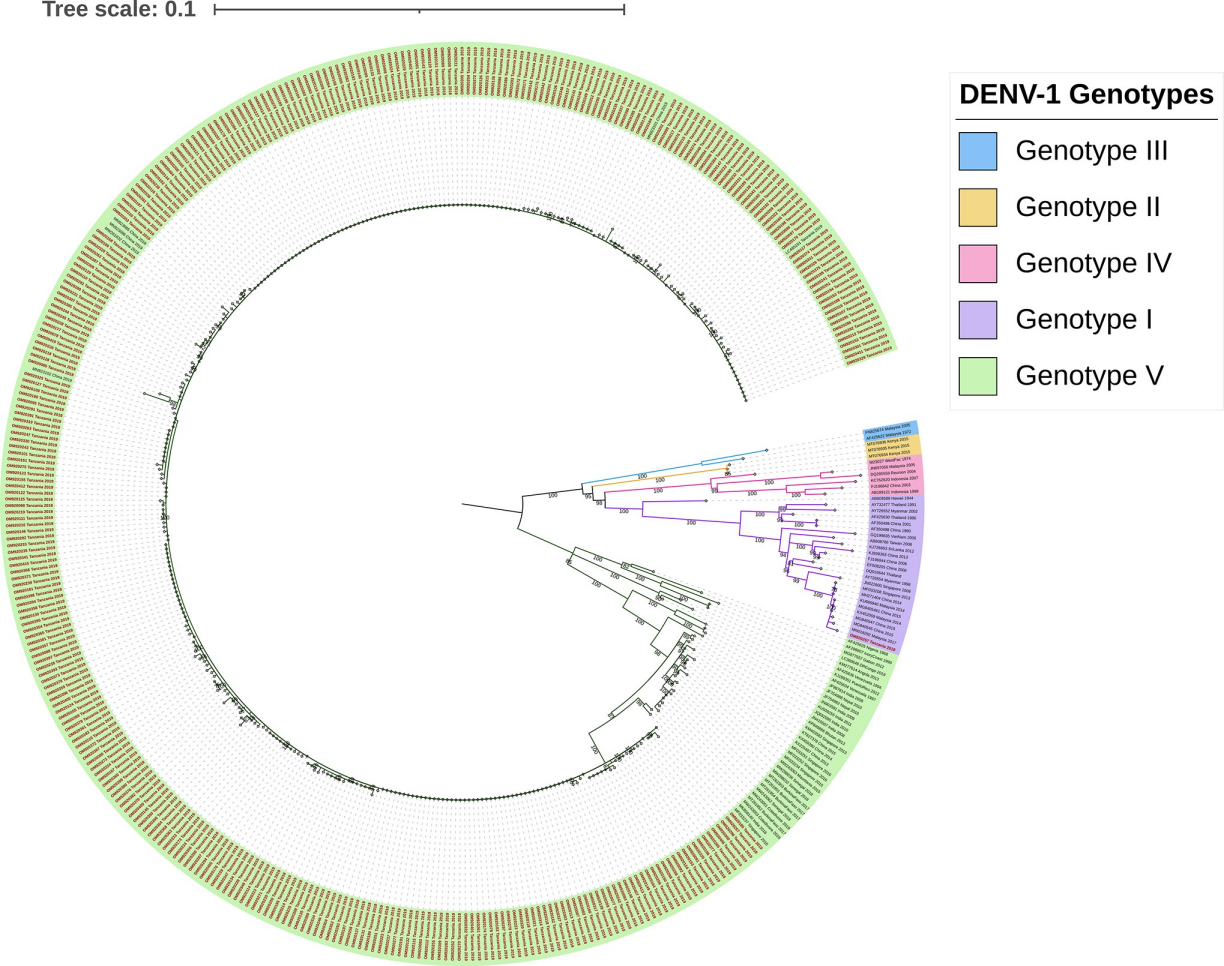
Genotyping of DENV-1 circulating in Tanzania in 2019. Maximum likelihood phylogenetic tree reconstructed with the 341 sequences generated by this study and 69 additional sequences from GenBank to provide genotype reference and geographic-temporal context. The tree was rooted at midpoint. Genotype I was only detected from one sample in 2019, while genotype V was found widely circulated in the 2019 epidemic. The Tanzanian sequences (OM920075—OM920415) in red. Genotypes are presented with colored highlighted branches; Genotypes IV, III, V, II, and I are highlighted in pink, blue, green, gold, and purple, respectively. Contextual sequences are labeled with GenBank accession number, country of origin, and year of isolation.

Fig 3 shows phylogenetic analysis of the 2019 DENV-1 epidemic in Tanzania. The analysis shows that the majority, 99.7% (340 of the 341), of the sequences cluster together, forming a monophyletic Tanzanian lineage within genotype V supported by 100% bootstrap replications. This 2019 Tanzanian DENV lineage is closely related to viruses circulating in Asia: sequence MF858140 isolated in India in 2016 and sequence MF033237 isolated in Singapore in 2015. Only one sequence, OM920257, (1/341) grouped within genotype I closely related to viruses circulating in Asia: sequence MG840545 isolated in China and sequences MN018292 and KX452059 isolated from Malaysia in 2015.

## Discussion

This study was conducted to characterize the serotypes and genotypes of DENV that circulated in Tanzania between 2017 and 2019 and to determine their geographic distribution. DENV-3 genotype III strains were found to be responsible for the sporadic cases reported in 2017 and 2018. However, the larger epidemic in 2019 was caused by DENV-1 genotype V. We found no evidence of co-circulation of serotypes between 2017 to 2019.

DENV-3 genotype III was first linked to Tanzania in 2010 when it was detected in a traveler returning to Japan from Tanzania [29]. Our study shows that the viruses sequenced in this study group within a clade different from the 2010 DENV strains, suggesting *in situ* viral evolution or the importation of a different strain, though it is difficult to determine the exact origin of the 2017 and 2018 dengue cases due to the unavailability of active surveillance in the country. In addition, the strains that circulated in Tanzania in 2017 and 2018 were phylogenetically related to those isolated in Pakistan in 2008.

The 2019 epidemic marked the first detection of DENV-1 transmission in Tanzania, indicating that a switch of circulating DENV serotype occurred sometime between 2018 and 2019. The transmission of DENV-1 may have been caused by importation since there is no evidence of prior circulation in the country; however, additional sampling of viruses within the time-period will be needed to determine the time and origin of the switch. Our study provides molecular evidence supporting that several shifts of the predominant circulating DENV serotype occurred in Tanzania between 2017–2019: whilst DENV-2 was mainly reported between 2010–2014 [24,30], DENV-3 replaced DENV-2 in 2017 and 2018, and followed by DENV-1 during the 2019 epidemic. Serotype switching could serve as a warning for the upcoming outbreak since there are high chances of having more severe cases after serotype switching [31].

As individuals can be infected with more than one serotype in their lifetime, the change in serotype increases the risk of life-threatening diseases such as dengue hemorrhagic fever and dengue shock syndrome, facilitated by antibody-dependent enhancement (ADE) [32,33]. As it has been observed that three different dengue virus serotypes caused several Tanzanian outbreaks within the last decade, we recommend implementing an active dengue surveillance system for early detection of dengue outbreaks and routinely monitoring the changes in serotypes and genotypes nationwide.

In this study, DENV-1 sequences from the 2019 epidemic formed several clusters within genotype V. The Tanzanian DENV-1 genotype V displayed high genetic similarity to sequences detected in Singapore in 2015 and India in 2016, suggesting introductions of these lineages before 2019. The Tanzania sequences also clustered together with DENV-1 genotype V sequences (GenBank: MN923101.1, and GenBank: MN923102.1) isolated from travelers returning to China from Tanzania in April and May 2019 respectively, implying that the individuals acquired the infection during their stay in Tanzania.

The foreign viruses closely related with the Tanzanian genotype V strains were identified during the dengue outbreak in Guangzhou in 2019. This shows the importance of international travel and global trade networks have the potential to spread DENV from one country to another or even between continents [29,34,35].

DENV-1 genotype I was also isolated from one case during the 2019 epidemic, indicating the possibility of circulating multiple genotypes during an outbreak. The genotype I sequence showed high similarity to sequences isolated in Malaysia in 2017 (MN018292). Since no clinical information was linked to this study, it was hard to assess if this particular genotype showed different clinical outcomes in a patient. There is a high necessity for active surveillance in the country with the integration of genomic surveillance.

All the samples tested in this study were collected from symptomatic cases during dengue outbreaks. There is a possibility that asymptomatic cases were left out and this may have underestimated the actual burden of the disease in our setting. In a study conducted in Dar es Salaam in 2020, less than 1% of suspected asymptomatic cases had dengue infection [36], significantly lower than the global WHO data that indicates over 80% of dengue infections are asymptomatic. This highlights the need for further investigation into this specific group of individuals in Tanzania.

Moreover, the study shows less than a 10% difference in positivity between males and females. The finding was consistent with earlier studies conducted in 2014 and 2018 [24,37], which also showed higher positivity in males who attended health facilities. One potential reason for this gender difference is that men may have fewer caregiving responsibilities, making it more convenient for them to seek medical attention when ill. However, it is important to note that we cannot conclude that men are inherently more susceptible to dengue infection than women.

Dar es Salaam was the epicenter of the dengue epidemic in 2019. Of the 823 positive cases, 92% of the patients were residents of Dar es Salaam. The remaining cases were detected in Tanga, Kilimanjaro, Coast, and Singida region. Tanzania experienced heavy rainfalls in 2019 compared to 2017 and 2018 where the precipitation caused floods that displaced people and damaged infrastructure in Dar es Salaam [38]. Copious precipitation favors a conducive environment for breeding *Aedes aegypti* mosquitoes, mostly in the water holding medium-sized plastic containers and tires [3,30,39,40]. Furthermore, Dar es Salaam is also Tanzania’s largest city and the country’s economic hub, putting the city at risk of high dengue transmission due to extensive business and economic activities between local and international traders. Moreover, the high population density of 3,100 people per square kilometer, increases the risk of dengue transmission among the people living in Dar es salaam [41]. Hence there is a need of having mosquito surveillance in the country since an increase in the mosquito population may increase the probability of vector-borne diseases.

The majority (73%) of the positive cases in Dar es Salaam city were residents of the Kinondoni district. The high dengue incidence in Kinondoni compared to other regions, like Ilala and Ubungo, is not surprising as it had also been reported in 2014 in a study to assess the risk of DENV transmissions in Dar es Salaam [30]. The district accounted for over half of the *Aedes aegypti* positive water-holding containers in 2014 [30]. Moreover, Kinondoni was found to have the highest house index and the largest adult mosquito density per trap [30]. Another study reported in 2018 also showed that Kinondoni residents could access health services early because of their awareness and knowledge compared to other districts like Ilala and Temeke [37].

The lack of dengue surveillance data between 2010–2019 to monitor dengue transmission and serotype circulation and the lack of regional dengue surveillance in the periphery of Tanzania to monitor dengue viruses coming in and out of the country presented a limitation to this study.

Also, all the facilities used a line list and laboratory forms that did not capture important epidemiological parameters like clinical features, high-risk occupational groups, and travel history. Therefore, we could not do further analysis. This study will open the door for further studies and sharpen the ministry of health data collection tool for further understanding the link between virological data and clinical information.

## Conclusion

This study revealed the DENV serotype switch and range of genotypes circulating in Tanzania during 2017–2019. DENV-1 genotype V and genotype I caused the 2019 dengue epidemic, whereas DENV-3 genotype III was responsible for the smaller outbreaks in 2017 and 2018. We highly recommend revising this study’s line list/ laboratory request forms to capture important information such as travel history, clinical features, and laboratory parameters.

The switching of serotypes could lead to more severe cases of DENV in the country. There is a need to strengthen dengue surveillance by having an active case surveillance system and incorporating complete genome sequence studies to study virus evolution in the country.

## Supporting information

S1 Table List of DENV-1 Tanzania sequences names, 2017–2019. (XLSX)

S2 Table List of DENV-3 Tanzania sequences names, 2017–2019. (XLSX)
